# Checklist of ichthyoplankton of NORI-D polymetallic nodule exploration claim (eastern Clarion-Clipperton Zone) during winter 2021

**DOI:** 10.3897/BDJ.13.e137744

**Published:** 2025-02-18

**Authors:** Leah A Bergman, Javier Montenegro, Charlotte A Seid, Tiffany S Bachtel, Frazer Mann, Erik V Thuesen, Dhugal J Lindsay, Jeffrey C Drazen

**Affiliations:** 1 Japan Agency for Marine-Earth Science and Technology, Yokosuka, Japan Japan Agency for Marine-Earth Science and Technology Yokosuka Japan; 2 Minderoo-UWA Deep-Sea Research Centre, School of Biological Sciences and Oceans Institute, The University of Western Australia, Perth, Australia Minderoo-UWA Deep-Sea Research Centre, School of Biological Sciences and Oceans Institute, The University of Western Australia Perth Australia; 3 University of California San Diego, La Jolla, United States of America University of California San Diego La Jolla United States of America; 4 National Oceanic and Atmospheric Administration (NOAA) Southwest Fisheries Science Center, La Jolla, United States of America National Oceanic and Atmospheric Administration (NOAA) Southwest Fisheries Science Center La Jolla United States of America; 5 Maersk Line, Ltd., Vancouver, Canada Maersk Line, Ltd. Vancouver Canada; 6 Evergreen State College, Olympia, United States of America Evergreen State College Olympia United States of America; 7 University of Hawaii at Manoa, Honolulu, United States of America University of Hawaii at Manoa Honolulu United States of America

**Keywords:** DNA barcoding, environmental baseline assessment, fish checklist, plankton survey, polymetallic nodule mining

## Abstract

**Background:**

There been increasing interest in polymetallic nodule mining within the Clarion-Clipperton Zone (CCZ). Polymetallic nodule mining within NORI-D will release a sediment plume within the water column and a previous mining collector test within the Nauru Ocean Resources Inc. (NORI-D) contract area released surface pollution from mining tailings. The mid-water plume, as well as accidental surface pollution, indicate that polymetallic nodule mining could impact surface plankton. Although the ichthyoplankton within the eastern tropical Pacific have been well-studied, recent data from within polymetallic nodule mining licence areas is lacking. Environmental Expedition C5e conducted an environmental baseline assessment of both pelagic and benthic fauna within the NORI-D region of the CCZ, which included the opportunistic collection of ichthyoplankton.

**New information:**

Ichthyoplankton were collected within NORI-D from November–December 2021 using two plankton nets and a Remotely Operated Vehicle (ROV). Here, we present a checklist of ichthyoplankton within the NORI-D licence area during this winter campaign. Eighteen samples were collected and identified through morphology, with a limited number identified through genetic sequencing. Specimens were from five orders, including Argentiniformes, Stomiiformes, Myctophiformes, Beloniformes and Scombriformes. This checklist will aid contractors and scientists conducting work within the CCZ to examine how wastewater discharge from polymetallic nodule mining could impact fish reproduction and ichthyoplankton survival.

## Introduction

The Clarion-Clipperton Zone (CCZ), an abyssal area in the northeast Pacific, is of particular economic interest due to the high abundance of seafloor polymetallic nodules which contain large quantities of manganese, nickel, copper, cobalt and rare earth elements (Hein et al. 2013, Hein et al. 2020). Nodule mining using tethered vehicles and initial at-sea mineral separation produces a sediment plume which can extend tens of kilometres from the original mining site (Spearman et al. 2020, Muñoz-Royo et al. 2022, Ouillon et al. 2022). Part of Annex 1 to ISBA/19/LTC/8, which provides recommendations on assessing environmental impacts within deep-sea mining areas, states that plankton communities in the upper 200 m should be characterised ‘if there is potential for surface discharge’ (International Seabed Authority 2015). During the pilot mining collector test in the NORI-D area of the eastern CCZ, there was overflow of water and nodule fragments onboard the collector ship (ISA Secretariat 2023, Yin et al. 2024). An initial assessment deemed that there was ‘no risk of serious harm to the marine environment from the event’ (ISA Secretariat 2023), yet this event indicates that there is potential for accidental surface waste discharge during polymetallic nodule mining operations.

Sediment released from polymetallic nodule mining contains copper, which is known to interefere with reproduction and survival across numerous taxa. In fishes, copper exposure has been shown to interfere with spermatogenesis, induce atresia and T4 and T3 hormone levels in the ovaries, inhibit spawning, reduce the mean diameter and weight of eggs, lower survival rate and slow hatchling growth (Benoit 1975, Kumar and Pant 1984, Johnson et al. 2007, James et al. 2008, Suvi et al. 2019). Copper can also accumulate within the reproductive organs (James et al. 2008), indicating that short-term exposure (96 hours) could affect reproduction and development even after leaving the impacted area. Other potential impacts to reproduction and larval survival from mining sediment release include reduced illumination due to turbidity, temperature shock from the release of cold bottom water and changes in salinity and oxygen levels (Matsumoto 1984).

To assess how polymetallic nodule mining and sediment release could impact fish populations, the ichthyoplankton in mining licence areas must be examined. Overall, the ichthyoplankton of the eastern tropical Pacific have been well-studied (Ahlstrom 1971, Ahlstrom 1972, Loeb 1984, León-Chávez et al. 2010). The majority of ichthyoplankton in the region are from the families Myctophidae and Phosichthyidae, with *Diogenichthyslaternatus* (Garman 1899) (family Myctophidae) and *Vinciguerrialucetia* (Garman 1899) (family Phosichthyidae) often dominating the ichthyoplankton captured in plankton net surveys and showing seasonal changes in abundance (Ahlstrom 1971, Ahlstrom 1972, Loeb 1984, León-Chávez et al. 2010). Although considerably less abundant, larvae from the family Scombridae (including the genus *Thunnus* South 1845) have also been collected from the eastern tropical Pacific (Ahlstrom 1971, Ahlstrom 1972, Matsumoto 1984, Reglero et al. 2014). The effects of deep-sea mining on nearby tuna fisheries, including affecting migratory routes and bioaccumulation of metals within muscle tissue, have been hypothesised as potentially major environmental impacts of polymetallic nodule mining (Van Der Grient and Drazen 2021, Amon et al. 2023, Tilot et al. 2024).

Although ichthyoplankton within the eastern tropical Pacific have already been characterised (Ahlstrom 1971, Ahlstrom 1972, Loeb 1984, León-Chávez et al. 2010), recent data with DNA barcoding from within polymetallic nodule licence areas is lacking. Here, we report the fish larvae and eggs collected during plankton net tows from the NORI-D area of the eastern Clarion-Clipperton Zone. Eighteen samples from NORI-D were opportunistically collected during November–December 2021 and identified through morphology, with eight supported by DNA sequences. Although limited, these data provide valuable information about which fishes are reproducing adjacent to or within this mining licence area, which can be used for future management considerations for this region.

## Materials and methods

### Location and Survey

**Figure 1.**
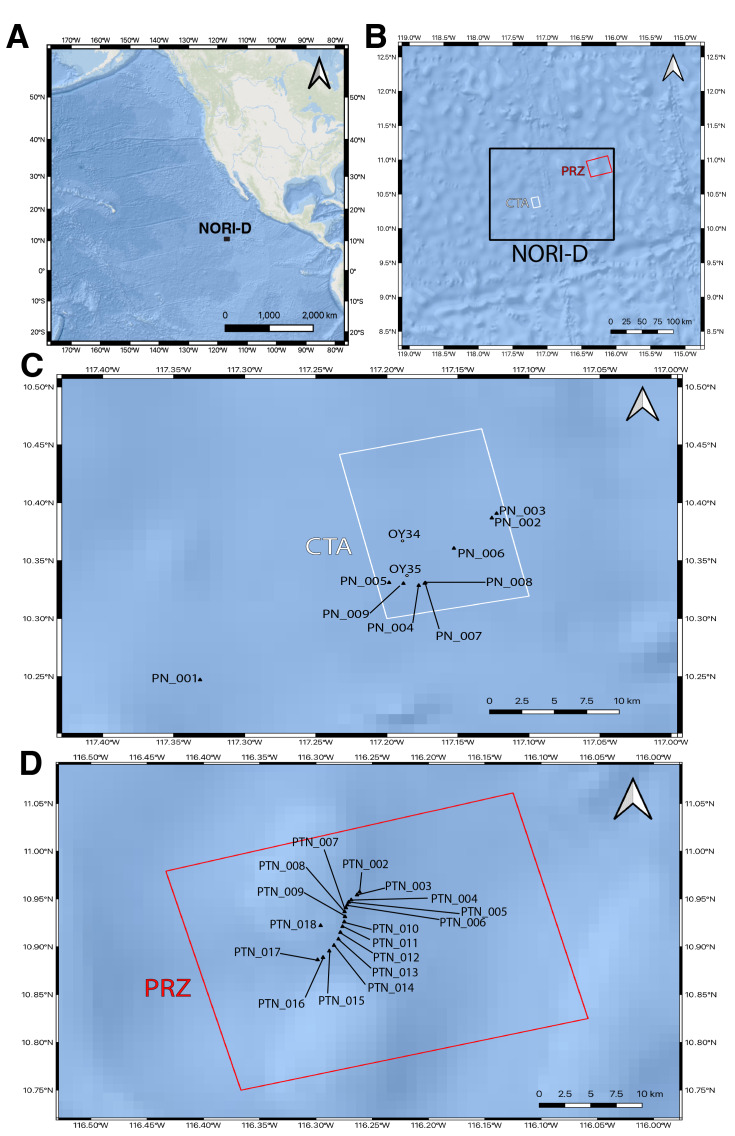
**A** Map of the eastern Clarion-Clipperton Zone, with NORI-D as a black polygon; **B** Map of NORI-D, with the Collector Test Area (CTA) and Preservation Reference Zone (PRZ) in white and red polygons, respectively; **C** Map of the CTA, with net tows (*PN* and *PTN*) shown in black triangles and ROV dives (*OY*) shown in white circles; **D** Map of the PRZ, with net tows (*PN* and *PTN*) shown in black triangles.

As part of an baseline environmental impact assessment conducted by The Metals Company Inc., the NORI-D mining licence area was surveyed by the *Maersk Launcher* in 2021. Two survey areas were sampled within NORI-D (Fig. 1), which include the Collector Test Area (CTA) and the Preservation Reference Zone (PRZ). The CTA is expected to be the site of seafloor polymetallic nodule mining, while the PRZ is a designated no-mining area. All specimens were opportunistically collected during Environmental Expedition C5e from 11 November though 19 December 2021.

Three different collection methods were used to capture fish larvae for this survey. They were conducted as a supplement to a formal environmental impact assessment (Table 1). The first method was a ring plankton net dubbed *PN* which was manually deployed and towed horizontally behind the ship. The mouth diameter was 20 cm with a mesh diameter of 330 μm and the cod end measured 3 cm diameter by 10 cm length. Nine surface tows were conducted in the CTA, which varied between 6.9 and 36.9 m wire out. A circular, open-mouthed plankton net dubbed *PTN* was used for vertical tows and deployed from the A-frame of the ship. The net had a mouth diameter of 70.5 cm with a mesh diameter of 330 μm and the cod end measured 30.8 long by 10.0 cm diameter. Seventeen tows were completed in the PRZ, all between 6:54–15:46 UTC on 16/12/2021 and varied between 50 and 226 m depth. Each tow spent 15–20 minutes at its target depth, with the maximum tow depth varying between 40 and 231 m. Some larvae were also accidentally captured in a concurrent ROV survey. The ROV *Odysseus* (Pelagic Research Services) dubbed *OY* was equipped with a 6-canister suction sampler with a variable-speed hydraulic pump. Each suction canister had an internal diameter of 152 mm and an internal height of 175 mm. The flexible hose through which specimens passed was 90 mm in internal diameter and the narrowest diameter in the piping system was 81 mm. The ROV was not aiming to sample larval fishes; therefore, all captures were incidentally collected by the sampling canister as bycatch during targeted sampling and their precise location and depth of capture were estimated.

### DNA Extraction and Identification

After collection, all samples were preserved in 99% ethanol and stored in -40℃. Images of the preserved samples were taken using a stereo dissecting microscope (Leica M165C) with a camera attachment (Canon EOS Kiss X7i with NY1S Micronet lens). Total DNA was extracted using the Promega Wizard ® HMW DNA Extraction Kit following the manufacturer's instructions, but adding 23 μl of sodium acetate (3 Molar at pH 5.2) and 3 μl of Ethachinmate (cat. 312-01791, FUJIFILM Wako) in step 11 to facilitate the DNA precipitation (Montenegro et al. 2023). Either metazoan or vertebrate CO1-mtDNA and 12S-rDNA primers were used to ensure the amplication of fish DNA.

CO1-mtDNA sequences were amplified using the universal metazoan primer set LCO1490 (5’-GGT CAA CAA ATC ATA AAG ATA TTGG-3’) and HCO2198 (5’-TAA ACT TCA GGG TGA CCA AAA AAT CA-3’) (Folmer et al. 1994), with the following PCR conditions; 5 cycles of 94°C x 30 sec, 47°C x 45 sec, 72°C x 1 min, 30 cycles of 94°C x 30 sec, 52°C x 45 sec, 72°C x 1 min and final extension of 72°C x 5 min. Positive amplifications were selected using 1.5% agarose electrophoresis and purified using Shrimp Alkaline Phosphatase (cat. 2660A, SAP, TaKaRa) and Exonuclease I (cat. 2650A, Exo-I, TaKaRa). The resulting amplicons were sent for Sanger sequencing to FASMAC Co. Ltd (Kanagawa, Japan).

12S-rDNA sequences were amplified using the vertebrate primer set 12SL (5’-AAA GCA CGG CAC TGA AGA TGC-3’) and 12SR (5’-TTT CAT GTT TCC TTG CGG TAC-3’) (Wang et al. 2000) with the following touchdown PCR conditions; 27 cycles of 94°C x 1 min, 46°C - 54°C x 1 min, 72°C x 1 min 30 sec and final extension of 72°C x 10 min. Pre-amplification of the primers above were tailed for nanopore sequencing and sequencing libraries were prepared following the protocol “SQK-LSK109 with EXP-PBC096” for amplicon sequencing with barcoding. The library was sequenced using a nanopore flow cell R9.4.1 and MinION Mk1C device. Resultant reads were basecalled using a Super Accurate model (SUP) and demultiplexed using Dorado v.0.7.2 (https://github.com/nanoporetech/dorado). The resultant reads were later filtered by size (1500 bp) and average quality (Q = 12) using Chopper v.0.8.0 (De Coster and Rademakers 2023; https://github.com/wdecoster/chopper). Finally, consensus sequences were generated per amplicon using the script amplicons_sorter (r2024/03/20) (Vierstraete and Braeckman 2022; https://github.com/avierstr/amplicon_sorter) with default settings. It is worth noting that despite multiple attempts to amplify the 12S-rDNA region using the standard MiFish-U-F/R primers (Miya et al. 2015), all PCR reactions were unsuccessful.

Fishes were visually identified using identification guides (Miller et al. 1979, Ahlstrom and Moser 1980, Moser 1996, Okayama 2014) prior to destruction for DNA extraction. DNA sequences were searched against publicly available sequences on the NCBI GenBank database using nucleotide BLAST (megablast algorithm) (Altschul et al. 1990, Zhang et al. 2000). The evaluation of BLAST matches is discussed below for each sample. Although there is no universal genetic metric for species delimitation and, even in fishes, there is no clearly delineated "barcoding gap" between intraspecific and interspecific variation (Ward 2009), commonly accepted thresholds for species-level identification are > 98% sequence identity for Cytochrome c oxidase I (COI) (Ward 2009, Ko et al. 2013, Zhang et al. 2021, Xing et al. 2022) and > 99% sequence identity for 12S (Milan et al. 2020). Specimens, their identities and GenBank accession numbers are given in the checklist below. An overview of the number of taxa and how they were identified is given in Table 2.

## Checklists

### Ichthyoplankton of NORI-D

#### Chordata

Haeckel, 1866

9C1389E6-2E83-501F-9925-D23E5930582C

#### Teleostei

Müller, 1845

A4CCEB29-A733-5183-9507-0D43B8DB8CAA

##### Materials

- **Type status:** Other material. **Occurrence:** catalogNumber: DL311; recordedBy: Leah A. Bergman; individualCount: 1; lifeStage: egg; otherCatalogNumbers: 211123z_6; occurrenceID: CCZ_NORID_C5e_DL311; **Location:** waterBody: Pacific Ocean; stateProvince: Clarion-Clipperton Zone; locality: The Metals Company Ltd exploration contract area (NORI-D); verbatimLocality: NORI-D, CTA; maximumDepthInMeters: 36.9; locationRemarks: Environmental Expedition C5e; decimalLatitude: 10.95477; decimalLongitude: -116.26357; geodeticDatum: WGS84; **Identification:** identifiedBy: Leah A. Bergman, Bruce C. Mundy; **Event:** samplingProtocol: PN; eventDate: 23/11/2021; eventTime: 20:56–21:04Z; fieldNumber: PTN_003; **Record Level:** collectionID: DL311
- **Type status:** Other material. **Occurrence:** catalogNumber: DL318; recordedBy: Leah A. Bergman; individualCount: 1; lifeStage: yolk-sac; otherCatalogNumbers: ML20211123SP-2; occurrenceID: CCZ_NORID_C5e_DL318; **Location:** waterBody: Pacific Ocean; stateProvince: Clarion-Clipperton Zone; locality: The Metals Company Ltd exploration contract area (NORI-D); verbatimLocality: NORI-D, CTA; maximumDepthInMeters: 36.9; locationRemarks: Environmental Expedition C5e; decimalLatitude: 10.387288; decimalLongitude: -117.126187; geodeticDatum: WGS84; **Identification:** identifiedBy: Leah A. Bergman, Bruce C. Mundy; **Event:** samplingProtocol: PN; eventDate: 23/11/2021; eventTime: 20:56–21:04Z; fieldNumber: PN_003
- **Type status:** Other material. **Occurrence:** catalogNumber: DL317; recordedBy: Leah A. Bergman; individualCount: 1; lifeStage: preflexion; otherCatalogNumbers: PITA_14-3; occurrenceID: CCZ_NORID_C5e_DL317; **Location:** waterBody: Pacific Ocean; stateProvince: Clarion-Clipperton Zone; verbatimLocality: NORI-D, PRZ; maximumDepthInMeters: 221; locationRemarks: Environmental Expedition C5e; verbatimCoordinateSystem: WGS84; decimalLatitude: 10.89612; decimalLongitude: -116.28816; geodeticDatum: WGS84; **Identification:** identifiedBy: Leah A. Bergman, Bruce C. Mundy; **Event:** samplingProtocol: PNT; eventDate: 16/12/2021; eventTime: 13:11–13:40Z; fieldNumber: PNT_015
- **Type status:** Other material. **Occurrence:** catalogNumber: DL326; recordedBy: Leah A. Bergman; individualCount: 1; lifeStage: preflexion; otherCatalogNumbers: SP20211209; occurrenceID: CCZ_NORID_C5e_DL326; **Location:** waterBody: Pacific Ocean; stateProvince: Clarion-Clipperton Zone; locality: The Metals Compary Ltd exploration contract area (NORI-D); verbatimLocality: NORI-D, CTA; maximumDepthInMeters: 6.9; locationRemarks: Environmental Expedition C5e; decimalLatitude: 10.330728; decimalLongitude: -117.188378; geodeticDatum: WGS84; **Identification:** identifiedBy: Leah A. Bergman, Bruce C. Mundy; **Event:** samplingProtocol: PN; eventDate: 30/11/2021; eventTime: 19:08–19:37Z; fieldNumber: PN_007
- **Type status:** Other material. **Occurrence:** catalogNumber: DL325; recordedBy: Leah A. Bergman; individualCount: 1; lifeStage: preflexion; otherCatalogNumbers: SP20211130-13; occurrenceID: CCZ_NORID_C5e_DL325; **Location:** waterBody: Pacific Ocean; stateProvince: Clarion-Clipperton Zone; locality: The Metals Compary Ltd exploration contract area (NORI-D); verbatimLocality: NORI-D, CTA; maximumDepthInMeters: 6.9; locationRemarks: Environmental Expedition C5e; decimalLatitude: 10.330728; decimalLongitude: -117.188378; geodeticDatum: WGS84; **Identification:** identifiedBy: Leah A. Bergman, Bruce C. Mundy; **Event:** samplingProtocol: PN; eventDate: 30/11/2021; eventTime: 19:08–19:37Z; fieldNumber: PN_007

##### Notes

Fig. 2

#### Argentiniformes

29F9A79E-6175-5B63-A8E5-23F0BA0526EE

##### Materials

- **Type status:** Other material. **Occurrence:** catalogNumber: DL314; recordedBy: Leah A. Bergman; individualID: 1; lifeStage: preflexion; otherCatalogNumbers: PITA_11-2; associatedSequences: GenBank (12S): PQ351602; occurrenceID: CCZ_NORID_C5e_DL314; **Location:** waterBody: Pacific Ocean; stateProvince: Clarion-Clipperton Zone; locality: The Metals Company Ltd exploration contract area (NORI-D); verbatimLocality: NORI-D, PRZ; maximumDepthInMeters: 200; locationRemarks: Environmental Expedition C5e; decimalLatitude: 10.91555; decimalLongitude: -116.2783; geodeticDatum: WGS84; **Identification:** identifiedBy: Leah A. Bergman, Bruce C. Mundy, Javier Montenegro; **Event:** samplingProtocol: PTN; eventDate: 16/12/2021; eventTime: 11:43–12:08Z; fieldNumber: PTN_012

##### Notes

Fig. 3

The closest 12S sequence match was 95.02% with *Lipolagusochotensis* (Schmidt 1938) family Bathylagidae (NC_004591.1). However, < 99% identity match is typically considered too broad for species-level identification (Milan et al. 2020, Kumar et al. 2022). Therefore, conservative identification to order Argentiniformes using morphology follows Bruce C. Mundy (July 2024, personal communication)

#### Stomiiformes

AFCA1D71-A805-519C-9B04-631F12F4BBFA

#### Gonostomatidae

Cocco, 1838

7A3BB697-355F-514C-A2D1-C96A4641DBFF

#### Cyclothone

Goode & Bean, 1883

5F53B6B4-449F-5A78-819F-018A313329D9

##### Materials

- **Type status:** Other material. **Occurrence:** catalogNumber: DL387; recordedBy: Leah A. Bergman; lifeStage: postflexion; otherCatalogNumbers: OY35SS1-1; 211208z-SS1-1; occurrenceID: CCZ_NORID_C5e_DL387; **Location:** waterBody: Pacific Ocean; stateProvince: Clarion-Clipperton Zone; locality: The Metals Company Ltd exploration contract area (NORI-D); verbatimLocality: NORI-D, CTA; minimumDepthInMeters: 75; maximumDepthInMeters: 1500; locationRemarks: Environmental Expedition C5e; decimalLatitude: 10.31731; decimalLongitude: -117.20236; geodeticDatum: WGS84; **Identification:** identifiedBy: Leah A. Bergman, Bruce C. Mundy; **Event:** samplingProtocol: ROV Odysseus; eventDate: 08/12/2021; fieldNumber: OY35

##### Notes

Fig. 4

#### Phosichthyidae

Weitzman, 1974

D3A458FD-7BE5-53D0-A041-170E42172FF9

#### Vinciguerria

Jordan & Evermann, 1896

CA9771B0-B4FB-5424-8356-27CBF5B01544

#### Vinciguerria lucetia

(Garman, 1899)

1D47DEED-0632-5E4D-8B66-98E4EA2ACAFA

##### Materials

- **Type status:** Other material. **Occurrence:** catalogNumber: DL320; recordedBy: Leah A. Bergman; lifeStage: flexion; otherCatalogNumbers: SP20211123Z-3; associatedSequences: GenBank: PQ327500; occurrenceID: CCZ_NORID_C5e_DL320; **Location:** waterBody: Pacific Ocean; stateProvince: Clarion-Clipperton Zone; locality: The Metals Company Ltd exploration contract area (NORI-D); verbatimLocality: NORI-D, CTA; maximumDepthInMeters: 37; locationRemarks: Environmental Expedition C5e; decimalLatitude: 10.39116; decimalLongitude: -117.12285; geodeticDatum: WGS84; **Identification:** identifiedBy: Leah A. Bergman, Bruce C. Mundy, Javier Montenegro; **Event:** samplingProtocol: PN; eventDate: 23/11/2021; eventTime: 20:56–21:04Z; fieldNumber: PN_003
- **Type status:** Other material. **Occurrence:** catalogNumber: DL321; recordedBy: Leah A. Bergman; individualCount: 1; lifeStage: preflexion; otherCatalogNumbers: SP20211125-5; associatedSequences: GenBank (CO1): PQ327501; occurrenceID: CCZ_NORID_C5e_DL321; **Location:** waterBody: Pacific Ocean; stateProvince: Clarion-Clipperton Zone; locality: The Metals Company Ltd exploration contract area (NORI-D); verbatimLocality: NORI-D, CTA; maximumDepthInMeters: 20; locationRemarks: Environmental Expedition C5e; decimalLatitude: 10.331383; decimalLongitude: -117.198422; geodeticDatum: WGS84; **Identification:** identifiedBy: Leah A. Bergman, Bruce C. Mundy, Javier Montenegro; **Event:** samplingProtocol: PN; eventDate: 25/11/2021; eventTime: 15:22Z; fieldNumber: PN_005
- **Type status:** Other material. **Occurrence:** catalogNumber: DL324; recordedBy: Leah A. Bergman; individualCount: 1; lifeStage: preflexion; otherCatalogNumbers: SP20211130-12; associatedSequences: GenBank (CO1): PQ327503; Genbank (12S): PQ351600; occurrenceID: CCZ_NORID_C5e_DL324; **Location:** waterBody: Pacific Ocean; stateProvince: Clarion-Clipperton Zone; locality: The Metals Company Ltd exploration contract area (NORI-D); verbatimLocality: NORI-D, CTA; maximumDepthInMeters: 7; locationRemarks: Environmental Expedition C5e; decimalLatitude: 10.33119; decimalLongitude: -117.172982; geodeticDatum: WGS84; **Identification:** identifiedBy: Leah A. Bergman, Bruce C. Mundy, Javier Montenegro; **Event:** samplingProtocol: PN; eventDate: 30/11/2021; eventTime: 19:08–19:37Z; fieldNumber: PN_007
- **Type status:** Other material. **Occurrence:** catalogNumber: DL322; recordedBy: Leah A. Bergman; individualCount: 1; lifeStage: flexion; otherCatalogNumbers: SP20211130-10; associatedSequences: GenBank (CO1): PQ327502; occurrenceID: CCZ_NORID_C5e_DL322; **Location:** waterBody: Pacific Ocean; stateProvince: Clarion-Clipperton Zone; locality: The Metals Company Ltd exploration contract area (NORI-D); verbatimLocality: NORI-D, CTA; maximumDepthInMeters: 7; locationRemarks: Environmental Expedition C5e; decimalLatitude: 10.33119; decimalLongitude: -117.172982; geodeticDatum: WGS84; **Identification:** identifiedBy: Leah A. Bergman, Bruce C. Mundy, Javier Montenegro; **Event:** samplingProtocol: PN; eventDate: 30/11/2021; eventTime: 19:08–19:37Z; fieldNumber: PN_007
- **Type status:** Other material. **Occurrence:** catalogNumber: DL313; recordedBy: Leah A. Bergman; individualCount: 1; lifeStage: flexion; otherCatalogNumbers: PITA_2-7; associatedSequences: GenBank (CO1): PQ327504; occurrenceID: CCZ_NORID_C5e_DL313; **Location:** waterBody: Pacific Ocean; stateProvince: Clarion-Clipperton Zone; locality: The Metals Company Ltd exploration contract area (NORI-D); verbatimLocality: NORI-D, PRZ; maximumDepthInMeters: 60; locationRemarks: Environmental Expedition C5e; decimalLatitude: 10.951607; decimalLongitude: -117.266673; geodeticDatum: WGS84; **Identification:** identifiedBy: Leah A. Bergman, Bruce C. Mundy, Javier Montenegro; **Event:** samplingProtocol: PTN; eventDate: 16/12/2021; eventTime: 07:18–07:38Z; fieldNumber: PNT_003
- **Type status:** Other material. **Occurrence:** catalogNumber: DL388; recordedBy: Leah A. Bergman; individualCount: 1; lifeStage: postflexion; otherCatalogNumbers: P20211216-2; occurrenceID: CCZ_NORID_C5e_DL388; **Location:** waterBody: Pacific Ocean; stateProvince: Clarion-Clipperton Zone; locality: The Metals Company Ltd exploration contract area (NORI-D); verbatimLocality: NORI-D, PRZ; maximumDepthInMeters: 60; locationRemarks: Environmental Expedition C5e; decimalLatitude: 10.951607; decimalLongitude: -117.266673; geodeticDatum: WGS84; **Identification:** identifiedBy: Leah A. Bergman, Bruce C. Mundy, Javier Montenegro; **Event:** samplingProtocol: PTN; eventDate: 16/12/2021; eventTime: 07:18–07:38Z; fieldNumber: PNT_003

##### Notes

Fig. 5

The COI sequences for five specimens (DL313, DL320, DL321, DL322, DL324) matched a reference sequence of *V.lucetia* (HQ010067, voucher SIO 09-204) with 100.0% identity and all other BLAST hits showed < 95% identity. DL388 was identified through morphology only (Moser 1996). This mesopelagic Pacific species (Fricke et al. 2024) has been previously recorded from Clipperton Atoll (Fourriére et al. 2014) and it is dominant in the larval fish assemblages of the eastern tropical Pacific off Mexico (León-Chávez et al. 2010). *V.lucetia* larvae are elongate and slender with ovoid eyes and usually possess a large melanophore ventrally near end of the caudal peduncle (Moser 1996).

#### Myctophiformes

4D44673F-9396-5F2F-9654-AEF24AAB82A0

#### Myctophidae

Gill, 1893

F2C39BE7-14AF-5130-9734-AA835419C6BF

#### Diogenichthys

Bolin, 1939

AABA6734-9842-5B8D-83FC-7947C3F2050C

#### Diogenichthys laternatus

(Garman, 1899)

5F333C7B-E3BB-55E9-BC08-E0A84AF64338

##### Materials

- **Type status:** Other material. **Occurrence:** catalogNumber: DL312; recordedBy: Leah A. Bergman; lifeStage: postflexion; otherCatalogNumbers: OY34-SS1; occurrenceID: CCZ_NORID_C5e_DL312; **Location:** waterBody: Pacific Ocean; stateProvince: Clarion-Clipperton Zone; locality: The Metals Company Ltd exploration contract area (NORI-D); verbatimLocality: NORI-D, CTA; minimumDepthInMeters: 75; maximumDepthInMeters: 1500; locationRemarks: Environmental Expedition C5e; decimalLatitude: 10.37227; decimalLongitude: -117.17892; geodeticDatum: WGS84; **Identification:** identifiedBy: Leah A. Bergman, Bruce C. Mundy, Javier Montenegro; **Event:** samplingProtocol: ROV Odysseus; eventDate: 06/12/2021; fieldNumber: OY34

##### Notes

Fig. 6

This abundant mesopelagic species occurs in the central and eastern Pacific (Evseenko 2006, Fricke et al. 2024). Adults have been recorded from Clipperton Atoll (Fourriére et al. 2014) and it is dominant in the larval fish assemblages of the eastern tropical Pacific off Mexico, Ecuador, Peru and Chile (Evseenko 2006, León-Chávez et al. 2010). *D.lanternatus* larvae are characterised by elliptical eyes, moderately-sized head and a melanophore on trunk above pre-anal arch of gut (Moser 1996).

#### Beloniformes

0A3CCCEA-AEDC-5C90-AA04-B0750CAE4FAA

#### Hemiramphidae

Gill, 1859

6C2B8E7B-9754-59DD-9553-D3A01E6EAE9C

#### Oxyporhamphus

Gill, 1864

6428D2F2-CA14-50AF-9BA3-67AADE763A9A

#### Oxyporhamphus micropterus

(Valenciennes, 1847)

322DEF83-F590-575F-BF34-EABBDA6B54A7

##### Materials

- **Type status:** Other material. **Occurrence:** catalogNumber: DL319; recordedBy: Leah A. Bergman; individualCount: 1; lifeStage: egg; otherCatalogNumbers: SP20211122T01300-5; associatedSequences: GenBank (CO1): PQ327499; occurrenceID: CCZ_NORID_C5e_DL319; **Location:** waterBody: Pacific Ocean; stateProvince: Clarion-Clipperton Zone; locality: The Metals Company Ltd exploration contract area (NORI-D); verbatimLocality: NORI-D, CTA; maximumDepthInMeters: 37; locationRemarks: Environmental Expedition C5e; decimalLatitude: 10.247537; decimalLongitude: -117.3303; geodeticDatum: WGS84; **Identification:** identifiedBy: Leah A. Bergman, Bruce C. Mundy, Javier Montenegro; **Event:** samplingProtocol: PN; eventDate: 22/11/2021; eventTime: 15:31–16:45Z; fieldNumber: PN_001
- **Type status:** Other material. **Occurrence:** catalogNumber: DL315; recordedBy: Leah A. Bergman; individualCount: 1; lifeStage: egg; otherCatalogNumbers: PITA_12-2; occurrenceID: CCZ_NORID_C5e_DL315; **Location:** waterBody: Pacific Ocean; stateProvince: Clarion-Clipperton Zone; locality: The Metals Company Ltd exploration contract area (NORI-D); verbatimLocality: NORI-D, PRZ; maximumDepthInMeters: 200; locationRemarks: Environmental Expedition C5e; decimalLatitude: 10.90867; decimalLongitude: -116.28005; geodeticDatum: WGS84; **Identification:** identifiedBy: Leah A. Bergman, Bruce C. Mundy; **Event:** samplingProtocol: PTN; eventDate: 16/12/2021; eventTime: 04:11–04:37Z; fieldNumber: PNT_013

##### Notes

Fig. 7

The COI sequence for DL319 was 99.83% identical to sequences of *Oxyporhamphusmicropterus* (Valenciennes 1847) (MZ892547.1; MZ050602.1, voucher FIFP2021-seq16; MZ028360.1), corroborating morphological identification of the egg. DL315 was identified, based on morphological similarity to DL319. This genus is widespread in the surface waters of the Atlantic, Pacific and Indo-Pacific Oceans, *O micropterus* occurring in Western Atlantic, Eastern Pacific and Indo-Pacific Oceans and *Oxyporhamphussimilis* Bruun 1935 occurring in Western and Eastern Atlantic Oceans (Collette 2004, Fricke et al. 2024). Adults have been recorded from Clipperton Atoll (Fourriére et al. 2014). Based on COI sequences, eggs and larvae have been recorded from the Ninety East Ridge of the eastern Indian Ocean (Zhang et al. 2021) and larvae have been recorded from Hawaiian waters (Xing et al. 2022). Eggs are characterised by short, numerous, pointed spines on the chorion (Ahlstrom and Moser 1980).

#### Scombriformes

7F235C85-3D05-57B7-861D-0831CDCEF140

#### Scombridae

Rafinesque, 1815

FE20913B-AC6F-56E8-BFD4-86AD8A308E95

#### Thunnus

South, 1845

55C7CAD8-D676-59A6-8F10-1D1E2C8FE009

##### Materials

- **Type status:** Other material. **Occurrence:** catalogNumber: DL323; recordedBy: Leah A. Bergman; lifeStage: preflexion; otherCatalogNumbers: SP20211130-11; occurrenceID: CCZ_NORID_C5e_DL323; **Location:** waterBody: Pacific Ocean; stateProvince: Clarion-Clipperton Zone; locality: The Metals Company Ltd exploration contract area (NORI-D); verbatimLocality: NORI-D, CTA; maximumDepthInMeters: 7; locationRemarks: Environmental Expedition C5e; decimalLatitude: 10.33119; decimalLongitude: -117.172982; geodeticDatum: WGS84; **Identification:** identificationID: Leah A. Bergman, Bruce C. Mundy; **Event:** samplingProtocol: PN; eventDate: 30/11/2021; eventTime: 11:08–11:38Z; fieldNumber: PN_007

##### Notes

Fig. 8

#### Gempylidae

Gill, 1862

292048AF-C48C-5EE9-AC1F-41202DCA78E9

#### Gempylus

Cuvier, 1829

49B4160C-C368-54F5-AD51-1BB67677F3F3

#### Gempylus serpens

Cuvier, 1829

0F680948-B169-56B5-ABBE-434BEBC318FF

##### Materials

- **Type status:** Other material. **Occurrence:** catalogNumber: DL316; recordedBy: Leah A. Bergman; lifeStage: preflexion; otherCatalogNumbers: PITA_13-4; associatedSequences: GenBank (CO1): PQ351601; occurrenceID: CCZ_NORID_C5e_DL316; **Location:** waterBody: Pacific Ocean; stateProvince: Clarion-Clipperton Zone; locality: The Metals Company Ltd exploration contract area (NORI-D); verbatimLocality: NORI-D, PRZ; maximumDepthInMeters: 200; locationRemarks: Environmental Expedition C5e; decimalLatitude: 10.90199; decimalLongitude: -116.28403; geodeticDatum: WGS84; **Identification:** identificationID: Leah A. Bergman, Bruce C. Mundy; **Event:** samplingProtocol: PTN; eventDate: 16/12/2021; eventTime: 04:43–05:10Z; fieldNumber: PTN_014

##### Notes

Fig. 9

This mesopelagic predatory species is widespread in tropical oceans (Choy et al. 2013, Mthethwa et al. 2023, Fricke et al. 2024). Its larvae are commonly captured nearshore in the Hawaiian Archipelago (Miller et al. 1979). Adults have been recorded from Clipperton Atoll (Fourriére et al. 2014). Based on COI sequences, eggs and larvae have been recorded from the Ninety East Ridge of the eastern Indian Ocean (Zhang et al. 2021) and larvae have been recorded from Hawaiian waters (Xing et al. 2022). G. serpens larvae are characterised by strong pre-opercular spines, well-developed dorsal and pelvic fin ray spines, with pigmentation on the brain, dorsal surface of the gut, the mid-body and along the body margin under the first dorsal fin (Moser 1996).

## Discussion

This report details ichthyoplankton opportunistically collected from November through December 2021 within a polymetallic nodule mining licence area in the eastern tropical Pacific. Of the three sampling methodologies from this report, the majority of samples were collected using *PN*, the net used for horizontal tows. Although the mouth diameter and cod end of *PN* were smaller than *PTN*, conducting horizontal tows near the surface (maximum wire out: 36.9 m) yielded more ichthyoplankton samples during the survey period. Consequently, more were collected from the CTA, where both *PN* and *OY* collected ichthyoplankton. Due to the CTA and PRZ being less than 100 km apart from each other, the difference in the amount of ichthyoplankton captured between the two regions was likely due to the difference in survey methodology.

One potential impact of polymetallic nodule mining within the Clarion-Clipperton Zone includes the release and suspension of copper. The majority of copper within seafloor sediment is confined to the upper 10 cm, with up to 120 ppm within the upper 20 cm of sediment in the eastern CCZ (Callender and Bowser 1980). In simulated polymetallic nodule mining experiments, the upper 15–20 cm of sediment were removed and resuspended (Vonnahme et al. 2020), indicating that sediment plumes may suspend a considerable amount of copper and have the potential to affect reproduction across taxa. Although more work is needed to quantify the impacts of polymetallic nodule mining, including modelling the impacts of sedimentation, trace metal release and shifts in the thermocline, creating an updated checklist of ichthyoplankton within polymetallic nodule mining licence areas is valuable for considering the potential impacts of mining on the fish community.

All taxa within this survey have been previously collected in the Clarion-Clipperton Zone and the eastern tropical Pacific, both as adults and larvae (Ahlstrom 1971, Ahlstrom 1972, Fourriére et al. 2014). The most abundant taxon within this survey was *Vinciguerrialucetia*, with six specimens captured. Previous work has also quantified this species as one of the most abundant in ichthyoplankton surveys within the region (Ahlstrom 1971, Ahlstrom 1972, Loeb 1984, León-Chávez et al. 2010). Adult *V.lucetia* comprise nearly 10% of the diet of tuna in the eastern tropical Pacific and are a key trophic link between the surface and the mesopelagic (Alverson 1963, Olson et al. 2014); therefore, any impact on their reproduction and development could affect a wide variety of taxa.

Although this survey only reported a single *Thunnus* sp. specimen and *Thunnus* sp. larvae are less common within the eastern tropical Pacific than *Vinciguerria* spp. (Ahlstrom 1971, Ahlstrom 1972, Matsumoto 1984), the presence of larval tuna within a polymetallic nodule mining licence area is noteworthy. This supports previous hypotheses suggesting that tuna are reproducing near polymetallic nodule licence areas and that polymetallic nodule mining could potentially impact their reproduction (Reglero et al. 2014, Van Der Grient and Drazen 2021). However, the importance of NORI-D as a spawning area for tuna remains unclear.

This report details eighteen ichthyoplankton samples captured within a polymetallic nodule mining licence area from November–December 2021. Several of these species also occur in recent DNA-based ichthyoplankton checklists from Hawaiian waters (Xing et al. 2022) and the eastern Indian Ocean (Zhang et al. 2021). The eastern Pacific sequences generated in this work thereby represent an important geographic data point for baseline studies of population connectivity. Sampling within NORI-D in this survey occurred only during wintertime, therefore more work is needed throughout the year to quantify seasonal shifts in fish reproduction. As polymetallic nodule mining is an emerging industry, the content of wastewater and sediment plumes is largely unknown. Examining both the contents and spread of mining discharge are critical in determining the impact polymetallic nodule mining will have on fish reproduction and ichtyoplankton survival.

## Supplementary Material

XML Treatment for Chordata

XML Treatment for Teleostei

XML Treatment for Argentiniformes

XML Treatment for Stomiiformes

XML Treatment for Gonostomatidae

XML Treatment for Cyclothone

XML Treatment for Phosichthyidae

XML Treatment for Vinciguerria

XML Treatment for Vinciguerria lucetia

XML Treatment for Myctophiformes

XML Treatment for Myctophidae

XML Treatment for Diogenichthys

XML Treatment for Diogenichthys laternatus

XML Treatment for Beloniformes

XML Treatment for Hemiramphidae

XML Treatment for Oxyporhamphus

XML Treatment for Oxyporhamphus micropterus

XML Treatment for Scombriformes

XML Treatment for Scombridae

XML Treatment for Thunnus

XML Treatment for Gempylidae

XML Treatment for Gempylus

XML Treatment for Gempylus serpens

## Figures and Tables

**Figure 2a.**
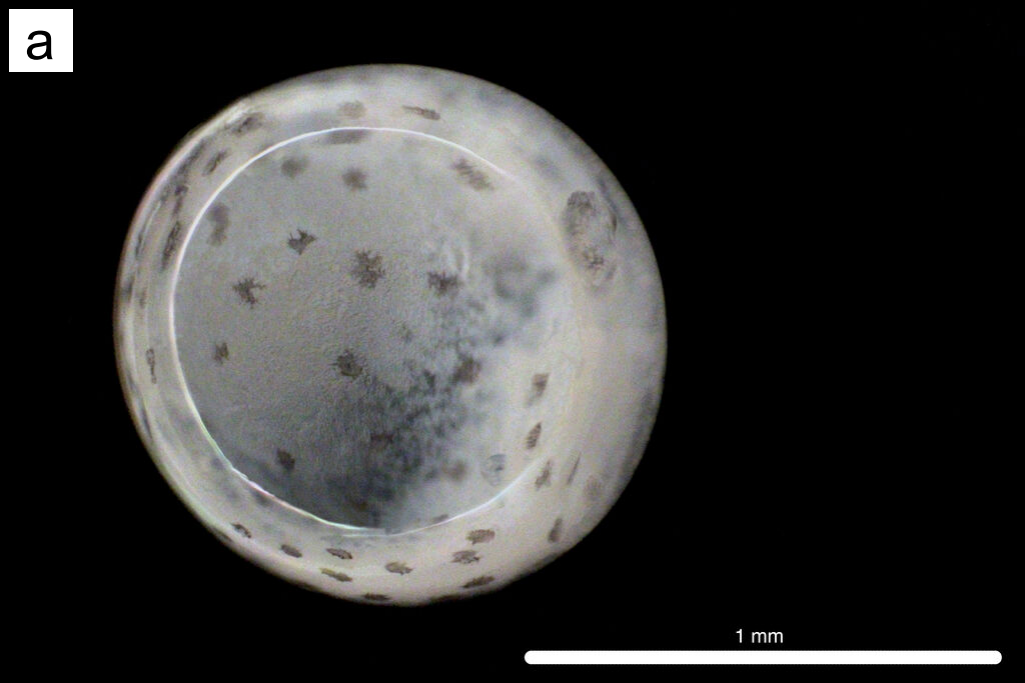


**Figure 2b.**
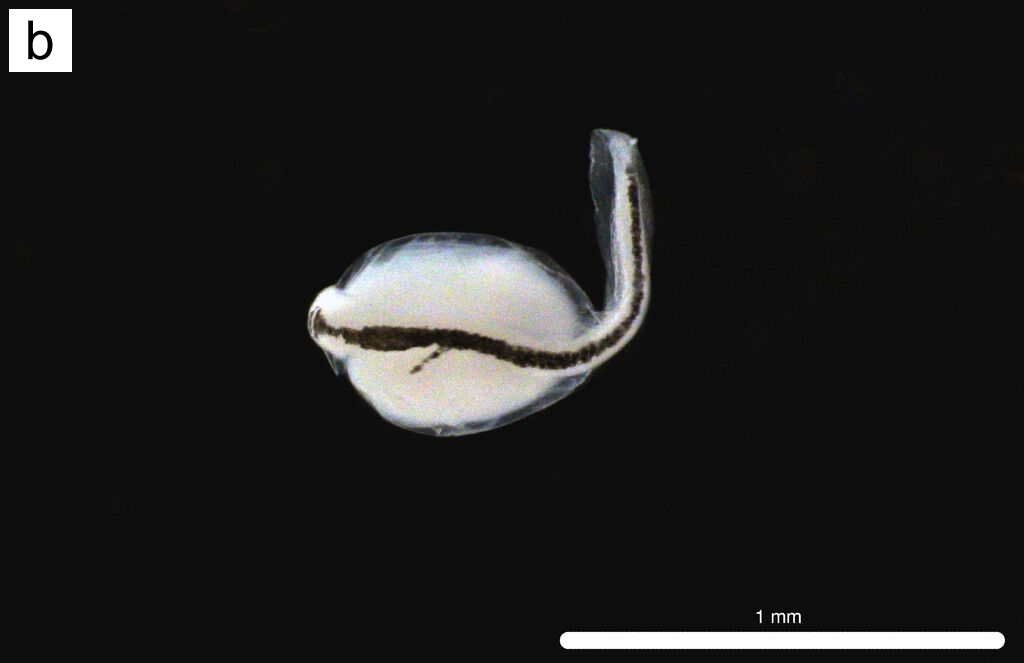


**Figure 2c.**
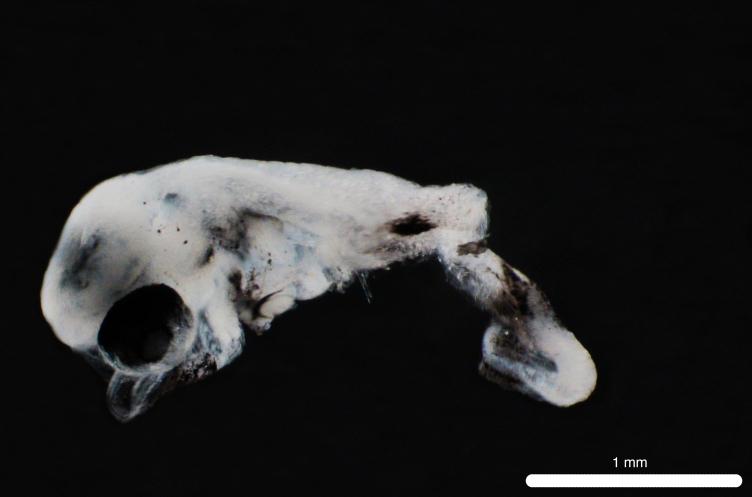


**Figure 2d.**
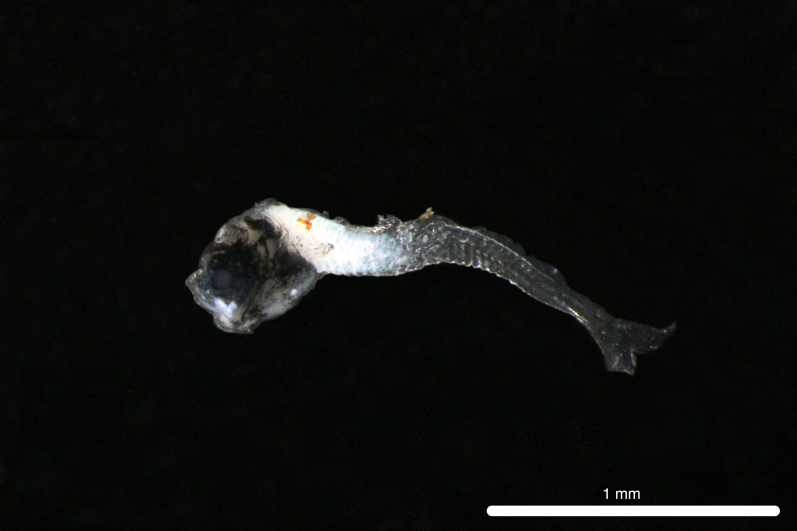


**Figure 2e.**
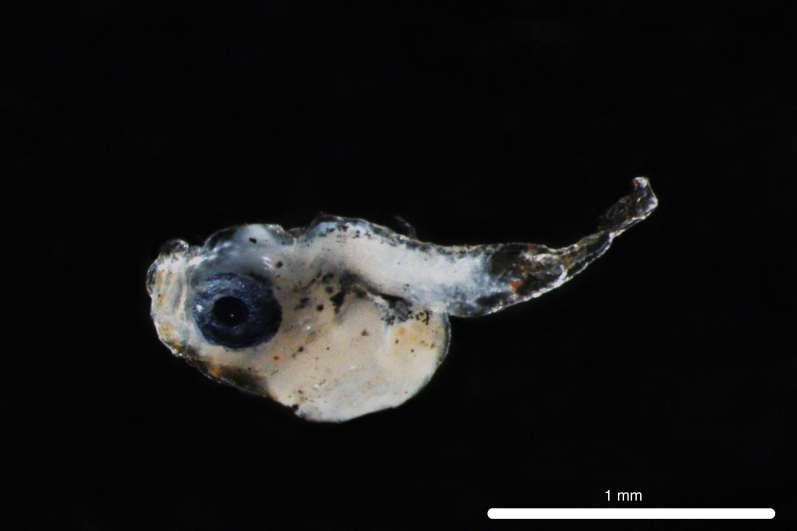


**Figure 3a.**
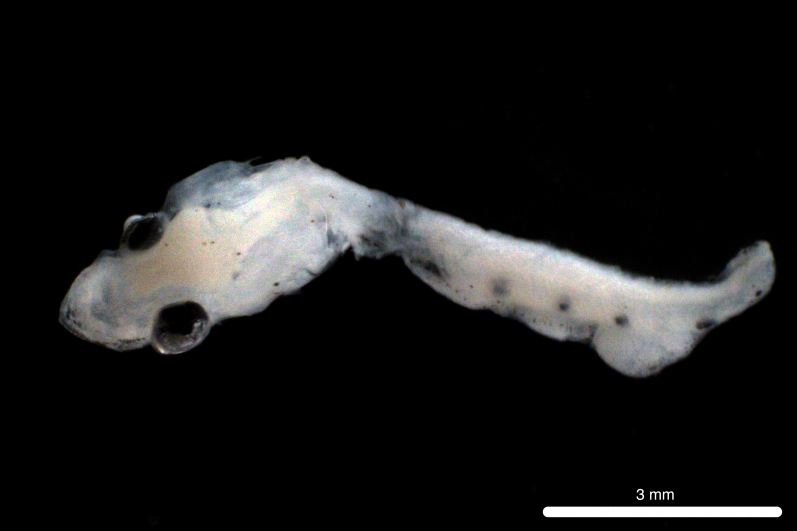


**Figure 3b.**
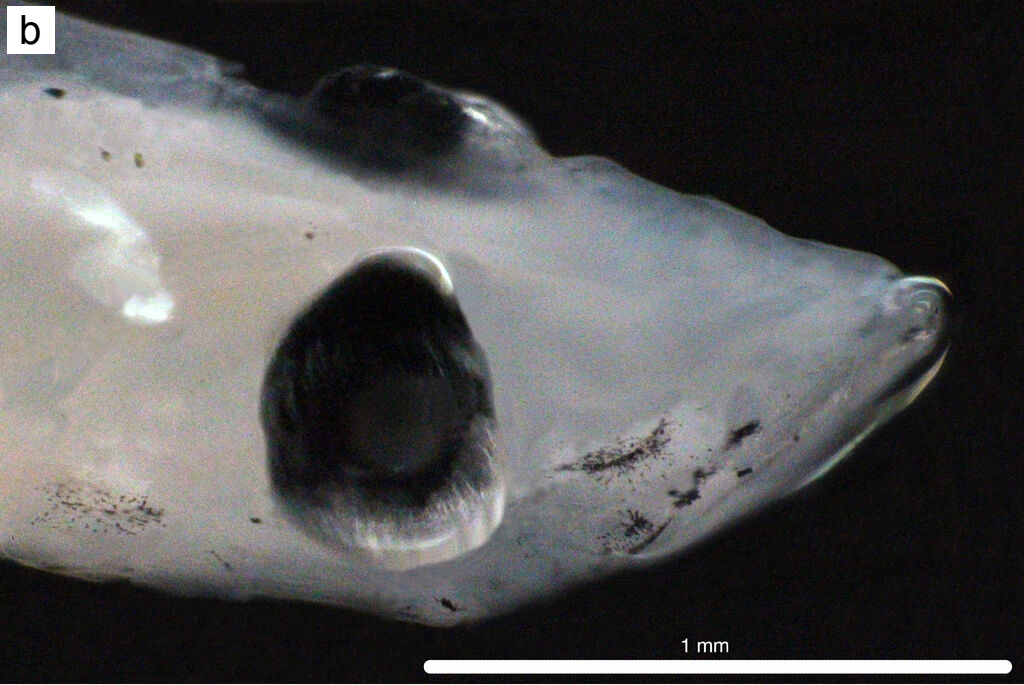


**Figure 4.**
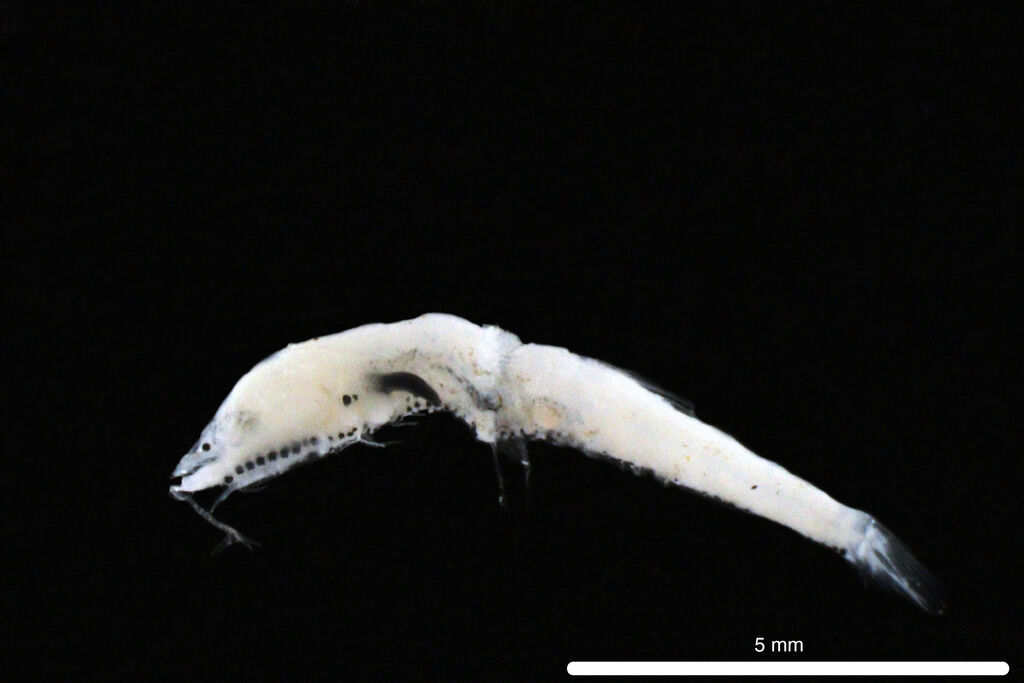
DL387 captured with the ROV *Odysseus* suction sampler during OY35.

**Figure 5a.**
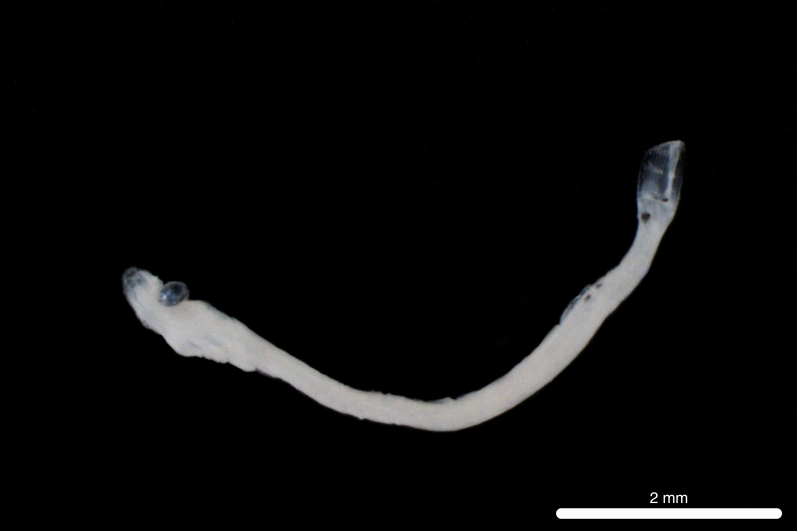


**Figure 5b.**
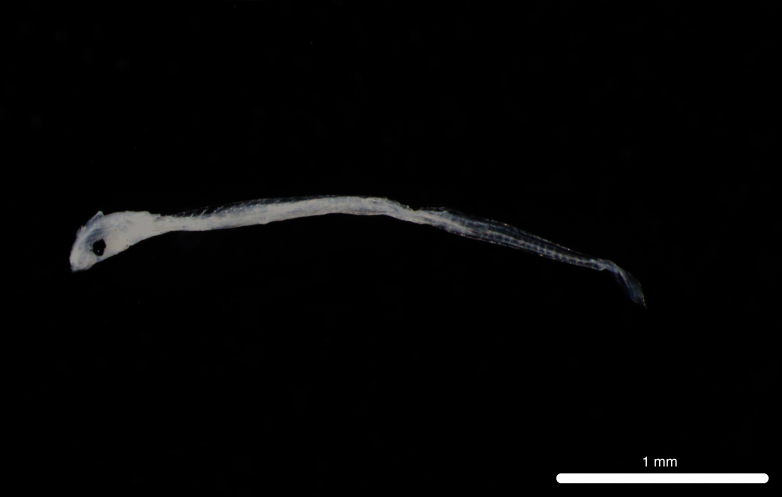


**Figure 5c.**
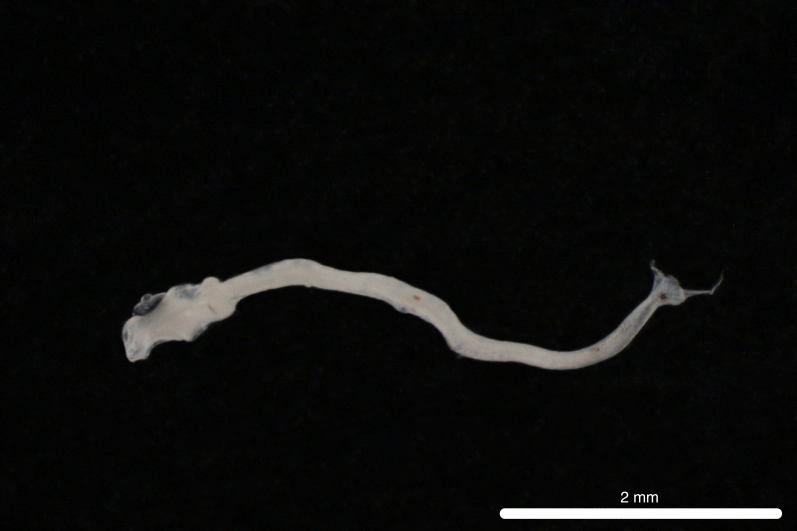


**Figure 5d.**
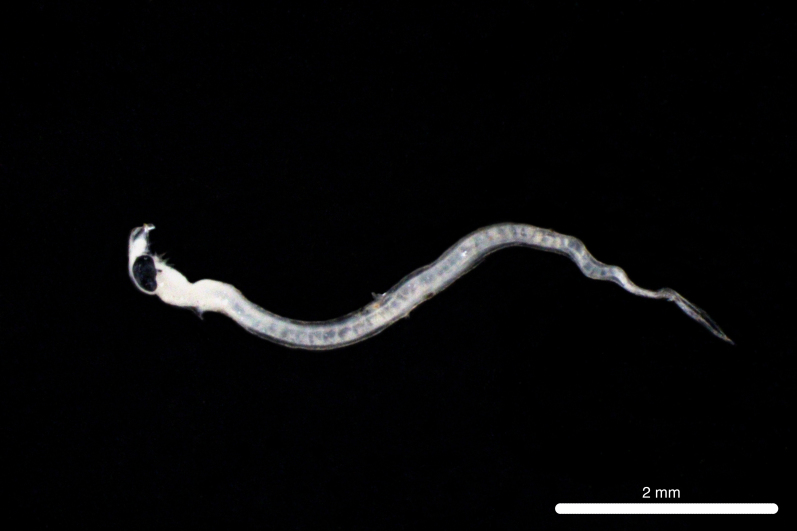


**Figure 5e.**
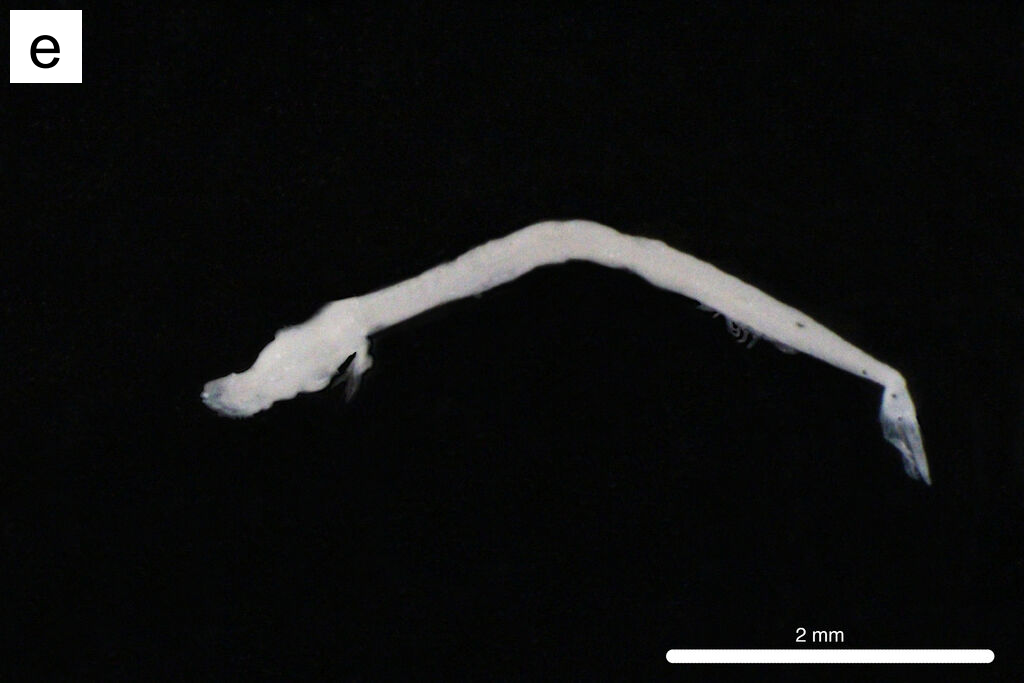


**Figure 5f.**
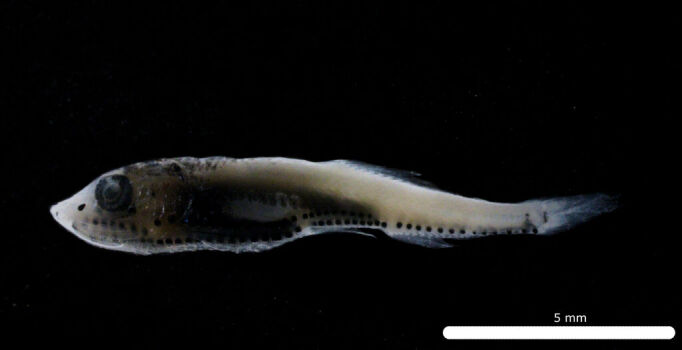


**Figure 6.**
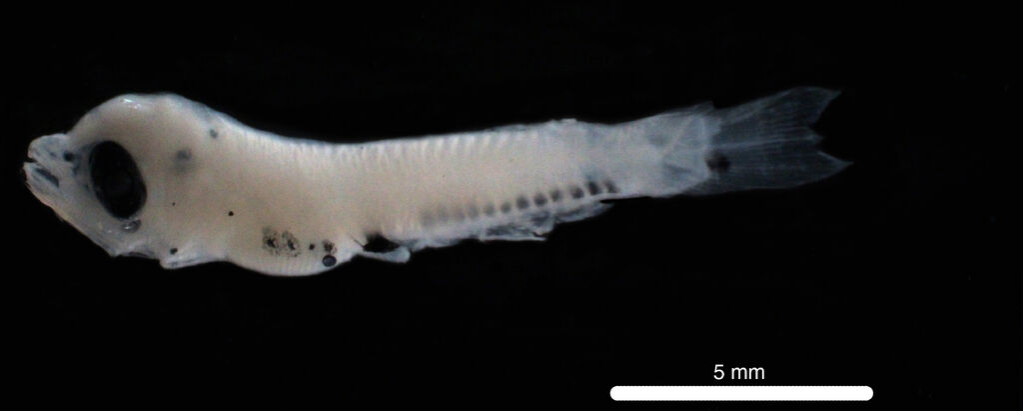
DL312 captured with the ROV *Odysseus* suction sampler during OY34.

**Figure 7a.**
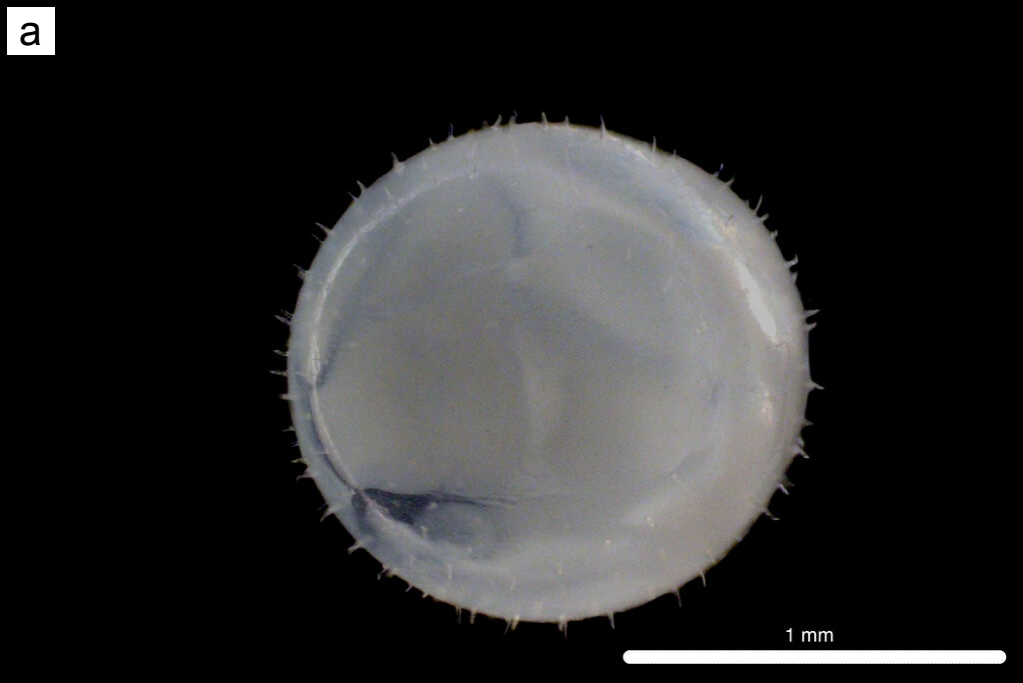


**Figure 7b.**
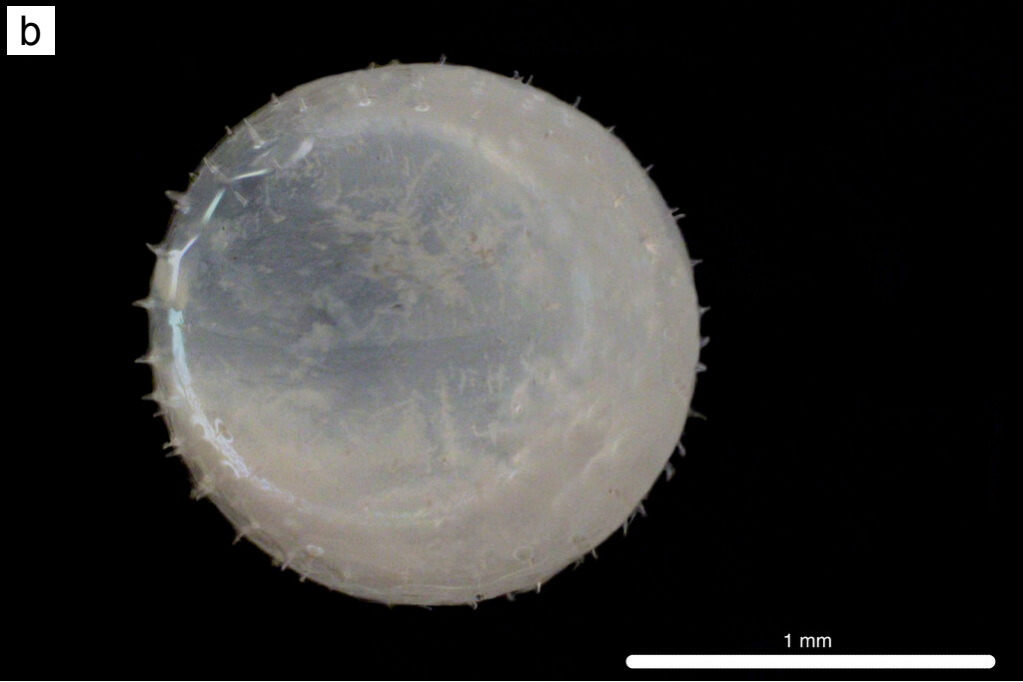


**Figure 8.**
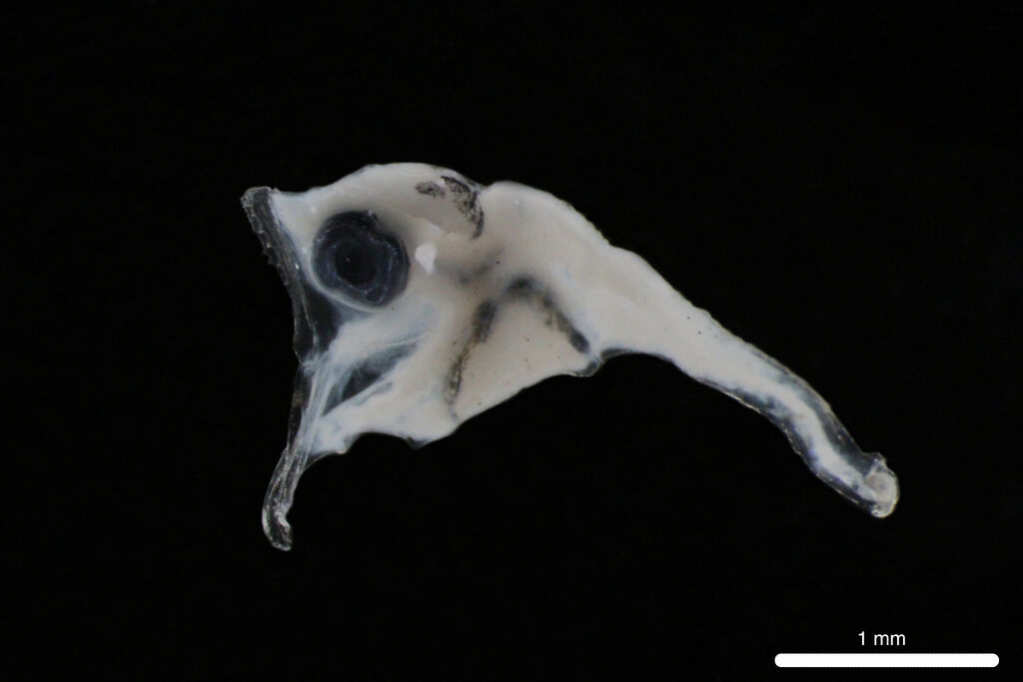
DL323 captured with PN in the CTA during PN_007.

**Figure 9.**
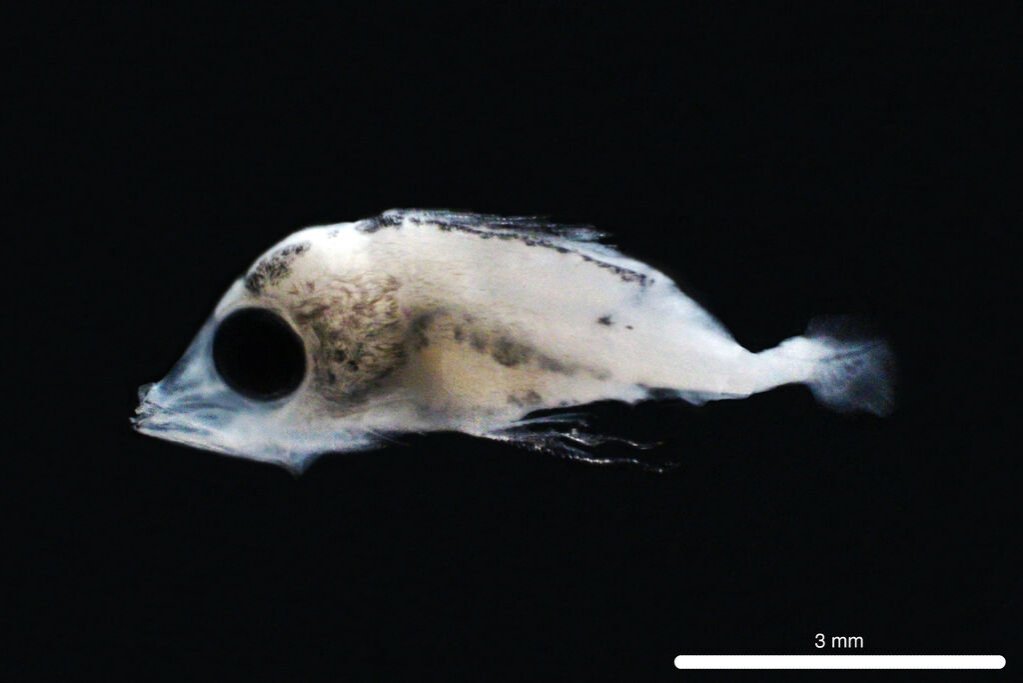
DL316 captured with PTN during PTN_014.

**Table 1.** Summary of plankton net tows and ROV dives where larval fishes were captured within NORI-D. The event (dive/tow number), event time (UTC datetime), decimal latitude, decimal longitude and the maximum depth in metres are given. Exact depth readings from an Ultra-short baseline (USBL) acoustic positioning beacon are indicated by *.

Event	Event Time	Decimal Latitude	Decimal Longitude	Maximum Depth in Metres
PN_001	22/11/2021T00:45–01:31Z	10.24754	-117.33147	36.9
PN_002	23/11/2021T19:32–20:32Z	10.38729	-117.12619	36.9
PN_003	23/11/2021T20:56–21:04Z	10.39116	-117.12285	36.9
PN_004	24/11/2021T02:50–03:04Z	10.32901	-117.17761	36.9
PN_005	25/11/2021T15:22Z	10.33147	-117.19842	19.9
PN_006	28/11/2021T21:11–21:30Z	10.36106	-117.15286	19.9
PN_007	30/11/2021T19:08–19:37Z	10.33085	-117.17343	6.9
PN_008	30/11/2021T23:00–23:27Z	10.33119	-117.17298	6.9
OY34	05/12/2021	10.36704	-117.18901	1500
OY35	06/12/2021	10.33715	-117.18589	1500
PN_009	09/12/2021T13:00Z	10.33073	-117.18839	6.9
PNT_002	16/12/2021T06:54–07:13Z	10.95791	-116.26127	40
PNT_003	16/12/2021T07:18–07:38Z	10.95477	-116.26357	60
PNT_004*	16/12/2021T08:02–08:26Z	10.94963	-116.26874	200
PNT_005*	16/12/2021T08:27–08:48Z	10.94725	-116.27097	100
PNT_006*	16/12/2021T08:52–09:14Z	10.94449	-116.27217	75
PNT_007*	16/12/2021T09:18–09:43Z	10.9414	-116.27346	50
PNT_008*	16/12/2021T09:44–10:11Z	10.93723	-116.27464	200
PNT_009*	16/12/2021T10:14–0:40Z	10.93209	-116.27428	200
PNT_010*	16/12/2021T10:42–11:07Z	10.92663	-116.27487	200
PNT_011*	16/12/2021T11:12–11:40Z	10.92172	-116.27643	200
PNT_012*	16/12/2021T11:43–12:08Z	10.91555	-116.2783	200
PNT_013*	16/12/2021T12:11–12:37Z	10.90867	-116.28005	218
PNT_014*	16/12/2021T12:43–13:10Z	10.90199	-116.28403	206
PNT_015*	16/12/2021T13:11–13:40Z	10.89612	-116.28816	221
PNT_016*	16/12/2021T13:44–14:07Z	10.88937	-116.29373	231
PNT_017*	16/12/2021T14:11–14:40Z	10.88694	-116.29856	226
PNT_018*	16/12/2021T15:31–15:46Z	10.9228	-116.2959	70

**Table 2.** Summary of specimens, including the total number collected, the number identified through morphology and the number identified through Cytochrome c oxidase I (COI) or 12S genetic sequencing.

Taxa	Number of specimens	Number from CTA	Number from PRZ	Identified from morphology	Identified from DNA sequencing
Unidentified Teleostei	5	4	1	5	0
Order Argentiniformes	1	0	1	1	1
*Cyclothone* sp. Goode & Bean, 1883	1	1	0	1	0
*Vinciguerrialucetia* (Garman 1899)	6	4	2	6	5
*Diogenichthyslanternatus* (Garman 1899)	1	1	0	1	0
*Oxyporhamphusmicropterus* (Valenciennes 1847)	2	1	1	2	1
*Thunnus* sp. South, 1845	1	1	0	1	0
*Gempylusserpens* Cuvier 1829	1	0	1	1	1
Total	18	12	6	18	8
